# The association between lower socioeconomic position and functional limitations is partially mediated by obesity in older adults with symptomatic knee osteoarthritis: Findings from the English Longitudinal Study of Ageing

**DOI:** 10.3389/fpubh.2022.1053304

**Published:** 2022-12-19

**Authors:** Rozemarijn Witkam, Suzanne M. M. Verstappen, James M. Gwinnutt, Michael J. Cook, Terence W. O'Neill, Rachel Cooper, Jennifer Humphreys

**Affiliations:** ^1^Centre for Epidemiology Versus Arthritis, Division of Musculoskeletal and Dermatological Sciences, The University of Manchester, Manchester, United Kingdom; ^2^NIHR Manchester Biomedical Research Centre, Manchester University NHS Foundation Trust, Manchester Academic Health Science Centre, Manchester, United Kingdom; ^3^Department of Sport and Exercise Sciences, Musculoskeletal Science and Sports Medicine Research Centre, Manchester Metropolitan University Institute of Sport, Manchester, United Kingdom; ^4^AGE Research Group, Translational and Clinical Research Institute, Faculty of Medical Sciences, Newcastle University, Newcastle upon Tyne, United Kingdom; ^5^NIHR Newcastle Biomedical Research Centre, Newcastle University and Newcastle upon Tyne Hospitals NHS Foundation Trust, Newcastle upon Tyne, United Kingdom

**Keywords:** socioeconomic position (SEP), obesity, functional limitations, joint replacement surgery, cohort study, ageing

## Abstract

**Objective:**

To assess the longitudinal associations of socioeconomic position (SEP) with functional limitations and knee joint replacement surgery (JRS) in people with symptomatic knee osteoarthritis (OA), and whether body mass index (BMI) mediated these relationships.

**Methods:**

Data came from the English Longitudinal Study of Ageing, a national longitudinal panel study of adults aged ≥50 years. A total of 1,499 participants (62.3% female; mean age 66.5 (standard deviation (SD) 9.4) years; 47.4% obese) self-reporting an OA diagnosis and knee pain, with at least one BMI measurement were included. Mixed effect models estimated longitudinal associations of each SEP variable (education, occupation, income, wealth and deprivation index) and obesity (BMI ≥30.0 kg/m^2^) with repeated measures of functional limitations. Cox regression analyses estimated associations between SEP indicators and obesity at baseline and risk of knee JRS at follow-up. Structural equation modeling estimated any mediating effects of BMI on these relationships.

**Results:**

Lower SEP and obesity at baseline were associated with increased odds of functional limitations in people with knee OA [e.g., difficulty walking 100 yards: no qualification vs. degree adjOR 4.33 (95% CI 2.20, 8.55) and obesity vs. no obesity adjOR 3.06 (95% CI 2.14, 4.37); similar associations were found for the other SEP indicators]. A small proportion of the association between lower SEP and functional limitations could be explained by BMI (6.2–12.5%). Those with lower income, lower wealth and higher deprivation were less likely to have knee JRS [e.g., adjHR most vs. least deprived 0.37 (95% CI 0.19, 0.73)]; however, no clear association was found for education and occupation. Obesity was associated with increased hazards of having knee JRS [adjHR 1.87 (95% CI 1.32, 2.66)]. As the direction of the associations for SEP and obesity with knee JRS were in opposite directions, no mediation analyses were performed.

**Conclusions:**

Lower SEP was associated with increased odds of functional limitations but lower hazards of knee JRS among people with knee OA, potentially indicating underutilization of JRS in those with lower SEP. Obesity partially mediated the relationship between lower SEP and increased odds of functional limitations, suggesting adiposity as a potential interventional target.

## Introduction

Globally, osteoarthritis (OA) is one of the leading causes of years lived with disability (1). Evidence has shown that there is a “discordance” between joint damage (measured through imaging) and symptomatic progression (measured through pain and disability questionnaires) in OA (2). Functional limitations, rather than structural changes, capture the impact of the disease on the day-to-day lives of people with OA (3). In addition, functional disability is an important predictor for mortality in people with OA (4).

There is currently no cure for OA. Therefore, the mainstay of treatment combines management of symptoms with pain relief, physiotherapy and, in end stage disease, joint replacement surgery (JRS) (5). Although JRS improves pain, function, and quality of life (6), joint replacements have a finite life expectancy and revision surgery may carry risks, such as infections (5). Understanding risk factors for functional limitations and JRS in people with OA is important as it allows physicians to monitor closely patients who are at increased risk for these adverse outcomes and identify factors that may modify this risk early in the disease process.

Socioeconomic position (SEP) refers to an individual's economic and social position within a society (7). Those with lower SEP have increased risk for OA (8) and a number of cross-sectional studies have found lower SEP to be associated with worse pain and function in people with OA (9, 10). However, recent research indicates that OA patients with lower SEP are less likely to undergo JRS than OA patients with higher SEP, even in tax-based healthcare systems where medical care is free at the point of use for everyone (11–13). This indicates that there may be an unmet need for JRS among those with lower SEP.

The relationship between lower SEP and worse disease progression may be mediated by obesity. Obesity is a well-known risk factor for the development of OA (14), and a recent prospective study indicated that body mass index (BMI) mediates the relationship between lower SEP and incident OA at any site (15). Although there is conflicting evidence about the relationship between obesity and radiographic progression of knee OA (16–18), recent systematic reviews indicated a strong association between BMI and symptomatic progression measured by pain and function (18), and weight loss resulted in symptomatic improvements (i.e., pain and function) in people with knee OA (19). Obese knee OA patients also have a higher need for knee JRS (20) and at a younger age (21) than non-obese knee OA patients. As the association between SEP and obesity is gender specific (22), the mediating effect of obesity for the relationship between SEP and OA disease progression may also differ by gender. Longitudinal studies are needed to understand how SEP and obesity interact in the progression of OA over time. This could be useful for risk stratification and to target obesity interventions to those who might benefit most.

Therefore, this study aimed to understand the relationships between SEP, obesity and symptomatic OA progression. The main research questions were (1) What are the longitudinal associations between SEP and functional limitations and knee JRS in people with symptomatic knee OA, and do they differ by gender or obesity status?; (2) What are the longitudinal associations between obesity and functional limitations and knee JRS in people with symptomatic knee OA, and do they differ by gender?; (3) Does BMI mediate the associations between a lower SEP and progression of symptomatic knee OA, and do they differ by gender?

## Methods

### Participants and study design

This study used data from the English Longitudinal Study of Ageing (ELSA), a national longitudinal panel study recording the health, social and economic circumstances of adults aged ≥50 years and their partners, living in private households in England (23). Data collection cycles (referred to as “waves”) occur every 2 years with data currently available for analysis for nine waves between 2002 and 2019. With consent an additional nurse visit was offered at waves 2, 4, 6, and 8 where a series of measurements (e.g., blood pressure, blood tests, anthropometric measurements) took place (24). Each wave aims to reassess all members of ELSA (regardless of how long they have been in the study), and collects data on newly recruited participants drawn from the Health Survey of England (HSE). The HSE is an annual cross-sectional study aiming to monitor the health of a representative sample of the English population. Written informed consent was obtained from all participants and ethical approval was acquired from the NHS Research Ethics Committees under the National Research and Ethics Service. The UK Data Service provided anonymized data for this study.

Symptomatic knee OA was defined using two questions asked at each wave. First, participants were asked “Has a doctor ever told you that you have (or had) any of the following conditions on this card?”. If “Arthritis” was chosen, they could indicate the type of arthritis (osteoarthritis, rheumatoid arthritis or some other kind of arthritis). A second question was used to specifically classify a patient as having knee OA: “Do you feel knee pain?” (does not specify a timeframe). If participants answered “yes” to this question in the same or a previous wave of the self-reported OA diagnosis, they were classified as having knee OA. Participants with at least one BMI measurement were included. Prevalent OA cases from wave 1 were excluded as we could not ascertain the self-reported date of diagnosis. Baseline assessment was defined as the first time participants reported having OA during waves 2–8. Supplementary Figure 1 shows the flowchart of sample selection for this study.

### Measurements/instruments

#### Exposure variables: Socioeconomic position and obesity at baseline

SEP was only assessed at baseline. The following categorical variables were used as indicators of SEP: highest qualification of education obtained (no qualifications, foreign/other; National Vocational Qualification (NVQ) 1/Certificate of Secondary Education (CSE) or other grade equivalent; NVQ2/General Certificate of Education (GCE) O-level equivalent (qualification normally obtained at age 16 in the UK); NVQ3/GCE A-level equivalent (qualification normally obtained at age 18 in the UK); higher education/below degree; NVQ4/NVQ5/degree or equivalent), current or most recent occupation classified using the UK National Statistics Socioeconomic Classification (NS-SEC)5 (25) (semi-routine occupations; lower supervisory and technical occupations; small employers and own account workers; intermediate occupations; managerial and professional occupations), household equivalised income fifths, household wealth fifths (includes non-housing and primary housing wealth minus debts) and relative deprivation fifths of small areas in England [based on the Index of Multiple Deprivation (IMD)] (26). The IMD is a measurement of relative deprivation of small areas in England based on seven categories of deprivation (income; employment; education, skills and training; health deprivation and disability; crime; barriers to housing and services; and living environment). The reference category for all socioeconomic indicators was the category representing the highest SEP group [i.e., having a degree, managerial and professional occupations, highest income fifth, highest wealth fifth and lowest (least deprived) IMD fifth].

Weight and height were measured by nurses in waves 2, 4, and 6 and by trained interviewers in wave 8. The BMI measurement closest to self-reported OA diagnosis was used. Obesity was defined as a BMI of 30 kg/m^2^ or higher. In the regression models, obesity (BMI ≥30.0 kg/m^2^) was compared with non-obesity (BMI <30.0 kg/m^2^).

#### Outcome variables: Functional limitations and joint replacement surgery

The first outcome was functional limitations, measured through five self-reported mobility indicators and the Activities of Daily Living (ADL), a self-reported physical capability questionnaire (27), at baseline and follow-up assessments. The five self-reported mobility indicators were recorded as binary variables (ability to perform the activity, yes/no), including: (1) walking 100 yards, (2) getting up from a chair after sitting for long periods, (3) climbing several flights of stairs without resting, (4) climbing one flight of stairs without resting, and (5) stooping, kneeling or crouching. Unlike ADL, which creates a validated score (27), the mobility indicators were not summed to avoid loss of information on specific mobility indicators. ADL comprises six activities, including dressing, walking across a room, bathing/showering, eating, getting in or out of bed and using the toilet. For each ADL, participants answered the question “because of a health or memory problem, do you have difficulty doing any of the activities on this card? Exclude any difficulties that you expect to last <3 months”, where participants could respond with yes or no. For this study, a continuous indicator of the number of ADLs where a participants reported “yes” was used. This resulted in a score from 0 to 6, where 0 is no difficulties and 6 is all difficulties present.

The second outcome measure was the first self-reported knee JRS due to arthritis at follow-up (waves 3–9). If participants answered “yes” to the question “whether right/left knee joint was replaced”, they were further asked what the reason for the knee replacement was (arthritis, fracture, other reason). If the answer was “arthritis”, it was recorded as knee JRS due to arthritis.

#### Covariates/additional variables

Data on covariates were collected at the baseline wave for each participant and were self-reported, including: gender (male, female), age (in years, continuous variable), ethnicity (white, non-white), smoking status (never smoked, ex-smoker, current smoker), and physical activity based on the classification used in the Allied Dunbar Survey of Fitness (28) (sedentary, low, moderate, high).

An adapted version of the Rheumatic Disease Comorbidity Index (RDCI) (29) was used to account for comorbid illness. All comorbid diseases comprising the RDCI were used [i.e., lung disease, cardiovascular disease, fracture, depression and cancer (all self-reported)], except for stomach ulcers, which are not recorded in ELSA. This resulted in a score from 0 to 8 (where 0 is no comorbidities and 8 the highest comorbidity score). NHS diabetes guidelines indicate that blood sugar levels need to be stable prior to performing surgery as peri-operative complications are more common in people with high blood sugar levels (30). Hence, it was decided to account for time-varying glycated hemoglobin (HbA1c) levels. HbA1c values were measured using nurse-collected blood samples in waves 2, 4, 6, and 8.

### Statistical analysis

#### Descriptive and longitudinal analysis

Baseline characteristics of the study sample were reported for categorical and continuous data using frequencies (%) and means with standard deviation (SD), respectively.

Linear mixed models (LMM) for continuous outcomes and generalized LMM for binary outcomes were used to estimate longitudinal associations between each SEP variable and repeated measures of functional limitations (adjusted for age and gender) and between obesity and repeated measures of functional limitations (adjusting for age, gender, SEP and RDCI). The association between SEP and functional limitations were only adjusted for age and gender as we did not want to adjust for any potential mediators. Mixed effects models take into account the within-person correlation across each participants' repeated measures.

Cox proportional hazards regression analyses estimated associations between each SEP variable and hazards of knee JRS (adjusting for age and gender) and for obesity and hazards of knee JRS (adjusting for age, gender, SEP, RDCI and time-varying HbA1C). Participants contributed person-time from baseline to either (a) date of the wave of knee JRS (the outcome), (b) loss to follow-up (including non-response and death), (c) end of follow-up (wave 9), whichever came first. As severe obesity (BMI >35) may be a contraindication for JRS, this association was tested for non-linearity using multivariable fractional polynomials (MFP). The proportional hazards assumption was tested using the Schoenfeld residuals test, where a *p*-value of < 0.05 indicates violation of the assumption. The assumption was fulfilled for all analyses.

To investigate whether the aforementioned associations differed by gender (or by SEP for the obesity analyses), interaction terms between obesity/SEP and gender and obesity and SEP were included in the models. If an interaction term was statistically significant (*p* ≤ 0.05), stratified analyses were performed.

Missing data were all <3.2%, except for wealth and income, which had 5.8% of missing values from the primary baseline sample of 1,499 (Table 1). The missing data was assumed to be missing at random (MAR). All independent variables with missing data were imputed using multiple imputations using chained equations (MICE) with 10 cycles. Analyses were performed in Stata v14 (StataCorp, College Station, TX).

**Table 1:** Baseline characteristics of the primary sample (*n* = 1,499) stratified by obesity status.

**Characteristics**	**With obesity** **(*n* = 711)**		**Without obesity** **(*n* = 788)**	
	**Frequencies (%)/mean (SD)**	**Missing**	**Frequencies (%)/mean (SD)**	**Missing**
Age, years	65.3 (8.8)	4 (0.6%)	67.7 (9.8)	8 (1.0%)
Gender, female	467 (65.7%)	0 (0.0%)	467 (59.3%)	0 (0.0%)
Ethnicity, white	682 (95.9%)	0 (0.0%)	759 (96.3%)	0 (0.0%)
**Education**		5 (0.7%)		4 (0.5%)
No qualification	267 (37.6%)		261 (33.1%)	
Other	75 (10.5%)		93 (11.8%)	
CSE/NVQ1	40 (5.6%)		34 (4.3%)	
O-level/NVQ2/GCE	139 (19.5%)		126 (16.0%)	
A-level/NVQ3	53 (7.5%)		58 (7.4%)	
Higher education/ <degree	72 (10.1%)		107 (13.6%)	
Degree/NVQ4/5	60 (8.4%)		105 (13.3%)	
**Occupation**		23 (3.2%)		21 (2.7%)
Semi-routine	303 (42.6%)		260 (33.0%)	
Lower supervisory/technical	90 (12.7%)		75 (9.5%)	
Small employers	65 (9.1%)		102 (12.9%)	
Intermediate	87 (12.2%)		100 (12.7%)	
Managerial/professional	143 (20.1%)		230 (29.2%)	
**Income fifths**		34 (4.8%)		53 (6.7%)
1: Lowest	168 (23.6%)		159 (20.2%)	
2	155 (21.8%)		178 (22.6%)	
3	149 (21.0%)		141 (17.9%)	
4	111 (15.6%)		141 (17.9%)	
5: Highest	94 (13.2%)		116 (14.7%)	
**Wealth fifths**		34 (4.8%)		53 (6.7%)
1: Lowest	210 (29.5%)		159 (20.2%)	
2	152 (21.4%)		158 (20.1%)	
3	128 (18.0%)		137 (17.4%)	
4	117 (16.5%)		140 (17.8%)	
5: Highest	70 (9.8%)		141 (17.9%)	
**Area-level deprivation fifths**		2 (0.3%)		3 (0.4%)
1: Most deprived	145 (20.4%)		121 (15.4%)	
2	152 (21.4%)		161 (20.4%)	
3	145 (20.4%)		164 (20.8%)	
4	140 (19.7%)		183 (23.2%)	
5: Least deprived	127 (17.9%)		156 (19.8%)	
**Smoking status**		1 (0.1%)		3 (0.4%)
Never smoked	248 (31.5%)		278 (35.3%)	
Ex-smoker	372 (47.2%)		382 (48.5%)	
Current smoker	90 (11.4%)		125 (15.9%)	
**Physical activity**		2 (0.3%)		0 (0.0%)
Sedentary	46 (6.5%)		46 (5.8%)	
Low	303 (42.6%)		236 (29.9%)	
Medium	281 (39.5%)		373 (47.3%)	
High	79 (11.1%)		133 (16.9%)	
RDCI comorbidities, two or more	353 (49.6%)	0 (0.0%)	350 (45.4%)	1 (0.0%)

CSE, Certificate of Secondary Education; GCE, General Certificate of Education; kg, kilograms; m, meters; NVQ, National Vocational Qualification; RDCI, rheumatic disease comorbidity index; SD, standard deviation.

As a sensitivity analysis, the aforementioned analyses were repeated in a larger sample that also included people with knee OA without a BMI measurement (*n* = 305). Using MICE, BMI was imputed in this sample at the time of OA diagnosis.

#### Mediation analysis

Structural equation modeling (SEM) using the Lavaan package in R was used to estimate the mediating effect of BMI on the relationship between SEP and functional limitations. The total effect of SEP on functional limitations can be divided into the indirect effect (i.e., effect mediated by BMI) and direct effect (i.e., effect independent of BMI).

Using confirmatory factor analysis, SEP was defined as a latent variable with education, occupation, wealth and income as observed indicators (the factor loading of IMD was non-significant (*p* < 0.05) and was therefore not included as an indicator). Mobility was defined as a latent variable with the five different indicators mentioned previously. Due to the unbalanced nature of our dataset (i.e., different number of time points for each observation), we were not able to use repeated measures in the SEM; therefore, average scores of both mobility and ADL were calculated.

Fit indices were used to assess the fit of the model, including comparative fit index (CFI) (≥0.95 indicates good fit), root mean square error of approximation (RMSEA) (≤0.08 indicates good fit) and standardized root mean square residual (SRMSR) (≤0.08 indicates good fit). The diagonally weighted least squares estimator (called ‘WLSMV' in Lavaan) was used as the SEP indicators were non-normally distributed ordinal variables (31). Confidence intervals around the indirect effects and the proportion mediated were calculated through bootstrapping. The analyses were adjusted for age, gender and number of follow-up waves. Analyses were also stratified by gender (adjusting for age and number of follow-up waves), as the association between SEP and obesity is gender specific (22).

## Results

### Description of the cohort

A total of 3,851 participants reported incident OA cases in waves 2–8 of ELSA. Of these, 1,804 (46.8%) reported knee pain on or before their OA diagnosis and were subsequently classified as having symptomatic knee OA. Of these, 1,499 (83.0%) had at least one BMI measurement; these participants comprised the primary baseline sample (Supplementary Figure 1). Of the primary sample, 711 (47.4%) were obese. The participants with obesity were slightly younger and had lower SEP (in terms of education, occupation, income, wealth and deprivation) compared with the participants without obesity (Table 1).

### The associations between socioeconomic indicators and functional limitations and knee joint replacement surgery in people with symptomatic knee OA

#### Functional limitations

A lower SEP (education, occupation, income, wealth and area-level deprivation) was associated with limitations in mobility (Table 2) and worse ADL scores (Table 3). For example, those with no qualification were more likely to have difficulties with walking 100 yards [adjOR 4.33 (95% CI 2.20, 8.55)] and had worse daily function based on ADL scores [adj regression-coefficient 0.31 (95% CI 0.11, 0.48)] compared with those with a degree.

**Table 2:** Generalized linear mixed model for the relationships of socioeconomic indicators and obesity with difficulties in mobility.

**Predictors**	**OR (95% CI) of reporting difficulty with each of the specified physical tasks**									
	**Walking 100 yards**		**Getting up from chair**		**Climbing several stairs**		**Climbing one stair**		**Stooping, kneeling, crouching**	
	**Unadjusted**	**Adjusted**	**Unadjusted**	**Adjusted**	**Unadjusted**	**Adjusted**	**Unadjusted**	**Adjusted**	**Unadjusted**	**Adjusted**
**Education**										
No qualification	6.06 (3.04, 12.07)	4.33 (2.20, 8.55)^†^	3.02 (2.01, 4.53)	3.07 (2.04,	8.37 (4.89, 14.34)	6.84 (4.01, 11.67)^†^^‡^	9.28 (5.35, 16.09)	6.69 (3.90, 11.49)^‡^	3.19 (2.01, 5.05)	2.91 (1.83, 4.63)^†^^‡^
4.63)^†^^‡^
Other	1.51 (0.65, 3.52)	1.29 (0.56, 2.97)^†^	1.93 (1.18, 3.16)	1.94 (1.19, 3.18)^†^	3.21 (1.68, 6.16)	2.93 (1.54, 5.57)^†^^‡^	3.10 (1.60, 5.98)	2.57 (1.35, 4.89)	1.97 (1.12, 3.45)	1.89 (1.08, 3.32)^†^^‡^
CSE/NVQ1	3.42 (1.19, 9.83)	2.66 (0.94, 7.52)^†^	2.95 (1.57, 5.56)	3.00 (1.58, 5.63)^†^	4.15 (1.80, 9.55)	3.55 (1.56, 8.11)^†^^‡^	3.27 (1.43, 7.50)	2.75 (1.22, 6.21)^‡^	2.33 (1.13, 4.82)	2.16 (1.05, 4.47)^†^^‡^
O-level/NVQ2/GCE	1.76 (0.82, 3.77)	1.72 (0.81, 3.63)^†^	2.33 (1.49, 3.64)	2.33 (1.49, 3.65)^†^	3.03 (1.69, 5.45)	3.00 (1.69, 5.35)^†^^‡^	2.65 (1.45, 4.83)	2.51 (1.40, 4.51)^‡^	1.93 (1.16, 3.20)	1.91 (1.15, 3.17)^†^^‡^
A-level/NVQ3	1.25 (0.49, 3.22)	1.39 (0.55, 3.53)^†^	1.88 (1.08, 3.26)	1.88 (1.08, 3.25)^†^	2.27 (1.10, 4.68)	2.34 (1.15, 4.78)^†^^‡^	1.94 (0.93, 4.05)	2.08 (1.01, 4.26)^‡^	1.28 (0.69, 2.38)	1.30 (0.70, 2.41)^†^^‡^
Higher education/ <degree	0.96 (0.42, 2.21)	0.92 (0.41, 2.09)^†^	1.73 (1.07, 2.80)	1.73 (1.07, 2.80)^†^	2.10 (1.12, 3.94)	2.02 (1.09, 3.76)^†^^‡^	1.37 (0.71, 2.63)	2.49)^‡^	1.52 (0.88, 2.61)	1.49 (0.87, 2.57)^†^^‡^
Degree/NVQ4/5	Ref	Ref	Ref	Ref	Ref	Ref	Ref	Ref	Ref	Ref
**Occupation**										
Semi-routine	4.44 (2.66, 7.43)	4.54 (2.74, 7.51)	2.00 (1.48, 2.70)	1.95 (1.44, 2.63)	3.37 (2.22, 5.10)	3.36 (2.23, 5.04)^†^	5.47 (3.65, 8.19)	5.29 (3.57, 7.84)	2.23 (1.58, 3.16)	2.14 (1.51, 3.03)
Lower supervisory/technical	3.39 (1.70, 6.80)	3.13 (1.59, 6.15)	1.57 (1.04, 2.38)	1.60 (1.05, 2.42)	1.95 (1.10, 3.45)	1.89 (1.08, 3.30)^†^	3.27 (1.89, 5.64)	3.25 (1.92, 5.50)	2.35 (1.43, 3.84)	2.38 (1.46, 3.90)
Small employers	2.46 (1.21, 5.00)	2.11 (1.05, 4.24)	1.63 (1.07, 2.49)	1.63 (1.07, 2.49)	2.06 (1.17, 3.64)	1.97 (1.12, 3.44)^†^	2.40 (1.39, 4.15)	2.21 (1.30, 3.75)	1.86 (1.15, 3.03)	1.84 (1.13, 2.98)
Intermediate	1.05 (0.52, 2.13)	1.13 (0.56, 2.28)	1.07 (0.72, 1.60)	1.02 (0.68, 1.53)	2.01 (1.18, 3.43)	2.05 (1.21, 3.45)^†^	1.88 (1.10, 3.19)	1.77 (1.05, 2.99)	1.44 (0.91, 2.28)	1.32 (0.83, 2.11)
Managerial/professional	Ref	Ref	Ref	Ref	Ref	Ref	Ref	Ref	Ref	Ref
**Income fifths**										
1: Lowest	8.92 (4.44, 17.93)	7.37 (3.70, 14.68)	2.25 (1.52, 3.33)	2.26 (1.53, 3.34)^†^	3.94 (2.32,6.67)	3.45 (2.04, 5.81)^†^^‡^	7.88 (4.61, 13.47)	6.39 (3.79, 10.78)	1.87 (1.19, 2.94)	1.71 (1.08, 2.69)^‡^
2	9.23 (4.61, 18.50)	6.34 (3.20, 12.57)	2.01 (1.37, 2.96)	2.02 (1.37, 3.00)^†^	5.09 (2.98, 8.69)	(2.35, 6.80)^†^^‡^	8.65 (5.10, 14.65)	6.40 (3.83, 10.70)	2.31 (1.46, 3.66)	2.09 (1.31, 3.31)^‡^
3	7.40 (3.57, 15.37)	5.32 (2.60, 10.91)	1.98 (1.31, 2.99)	1.99 (1.31, 3.01)^†^	3.48 (2.01, 6.00)	2.86 (1.66, 4.92)^†^^‡^	6.19 (3.57, 10.73)	4.64 (2.71, 7.93)	2.25 (1.40, 3.60)	2.04 (1.27, 3.27)^‡^
4	2.26 (1.08, 4.76)	1.94 (0.93, 4.03)	1.41 (0.93, 2.14)	1.41 (0.93, 2.15)^†^	1.77 (1.03, 3.07)	2.80)^†^^‡^	2.46 (1.41, 4.29)	2.21 (1.29, 3.81)	1.34 (0.82, 2.19)	1.30 (0.80, (0.80, 2.11)^‡^
5: Highest	Ref	Ref	Ref	Ref	Ref	Ref	Ref	Ref	Ref	Ref
**Wealth fifths**										
1: Lowest	36.11 (18.07, 72.15)	36.50 (18.60, 71.63)^†^	3.81 (2.60, 5.60)	3.79 (2.58, 5.56)	11.96 20.33)	12.05 (7.17, 20.25)^†^^‡^	21.74 (12.82, 36.86)	21.63 (13.01, 35.96)	4.43 (2.81, 6.97)	4.38 (2.79, 6.87)
2	15.64 (7.78, 31.45)	14.93 (7.57, 29.44)^†^	2.35 (1.60, 3.45)	2.33 (1.59, 3.42)	5.12 (3.02, 8.66)	4.95 (2.97, 8.27)^†^^‡^	8.07 (4.76, 13.68)	7.51 (4.52, 12.45)	2.92 (1.86, 4.58)	2.85 (1.83, 4.45)
3	5.06 (2.45, 10.45)	4.48 (2.21, 9.07)^†^	1.73 (1.16, 2.57)	1.71 (1.15, 2.54)	2.87 (1.68, 4.91)	2.64 (1.56, 4.48)^†^^‡^	4.06 (2.36, 6.98)	3.61 (2.14, 6.08)	1.92 (1.20, 3.07)	1.83 (1.14, 2.91)
4	3.43 (1.64, 7.15)	3.20 (1.58, 6.54)^†^	1.37 (0.91, 2.04)	1.35 (0.90, 2.02)	2.25 (1.30, 3.88)	3.67)^†^^‡^	2.58 (1.48, 4.51)	2.38 (1.39, 4.07)	1.80 (1.12, 2.90)	1.74 (1.09, (1.09, 2.78)
5: Highest	Ref	Ref	Ref	Ref	Ref	Ref	Ref	Ref	Ref	Ref
**Area-level deprivation fifths**										
5: Most deprived	8.44 (4.42, 16.13)	11.55 (6.11, 21.82)^†^	2.42 (1.65, 3.56)	2.48 (1.69, 3.65)	3.31 (1.94, 5.65)	4.00 (2.34, 6.73)^†^^‡^	6.33 (3.81, 10.50)	8.20 (5.00, 13.43)^†^^‡^	2.52 (1.59, 3.99)	2.78 (1.76, 4.40)
4	3.86 (2.08, 7.19)	4.83 (2.63, 8.86)^†^	1.65 (1.15, 2.37)	1.69 (1.17, 2.43)	1.80 (1.09, 2.98)	2.05 (1.25, 3.35)^†^^‡^	3.56 (2.19, 5.79)	4.28 (2.67, 6.86)^†^^‡^	1.33 (0.87, 2.03)	1.44 (0.94, 2.20)
3	1.45 (0.77, 2.73)	1.62 (0.89, 2.99)^†^	1.04 (0.72, 1.49)	1.05 (0.73, 1.51)	1.18 (0.72, 1.94)	1.25 (0.77, 2.04)^†^^‡^	1.75 (1.07, 2.86)	1.92 (1.20, 3.09)^†^^‡^	1.19 (0.78, 1.83)	1.25 (0.82, 1.90)
2	1.76 (0.95, 3.27)	1.93 (1.06, 3.52)^†^	1.31 (0.91, 1.87)	1.32 (0.92, 1.89)	1.46 (0.89, 2.40)	1.54 (0.95, 2.50)^†^^‡^	2.14 (1.32, 3.46)	2.31 (1.45, 3.68)^†^^‡^	1.45 (0.95, 2.20)	1.50 (0.98, 2.27)
1: Least deprived	Ref	Ref	Ref	Ref	Ref	Ref	Ref	Ref	Ref	Ref
**Obesity**										
Obesity	3.51 (2.37, 5.20)	3.06 (2.14, 4.37)	2.06 (1.63, 2.59)	1.74 (1.39, 2.19)	3.92 (2.86, 5.37)	3.21 (2.40, 4.28)	3.18 (2.35, 4.31)	2.68 (2.05, 3.52)	2.77 (2.11, 3.63)	2.39 (1.83, 3.12)
Non-obesity	Ref	Ref	Ref	Ref	Ref	Ref	Ref	Ref	Ref	Ref
BMI per 1 kg/m^2^ increment	1.14 (1.10, 1.17)	1.13 (1.09, 1.16)	1.08 (1.06, 1.10)	1.06 (1.04, 1.08)	1.56 (1.12, 1.19)	1.14 (1.11, 1.16)	1.12 (1.09, 1.14)	1.11 (1.08, 1.13)	1.12 (1.10, 1.15)	1.11 (1.08, 1.14)

BMI, body mass index; CI, confidence interval; NVQ, National Vocational Qualification; OR, odds ratio; RDCI, rheumatic disease comorbidity index; ref, reference category. SEP indicators adjusted for age and gender. Obesity/BMI adjusted for age, gender, SEP and RDCI.

† Significant interaction with gender (0.001>p<0.05); therefore these estimates were only adjusted for age.

‡ Significant interaction between SEP and obesity (0.01>p<0.05). Stratified analyses for gender and obesity are shown in Supplementary Tables 2, 3. No evidence of interaction between obesity and gender (0.09 > *p*<0.87).

**Table 3:** Linear mixed effects models for the relationships of socioeconomic indicators and obesity with difficulties in activities in daily living score (0–6, 0 = no difficulties).

**Predictors**	Regression coefficient^*^(95% CI)	
	**Unadjusted**	**Adjusted**
**Education**		
No qualification	0.36 (0.17, 0.54)	0.31 (0.11, 0.48)^†^
Other	0.06 (−0.17, 0.28)	0.03 (−0.20, 0.25)^†^
CSE/NVQ1	0.23 (−0.07, 0.52)	0.18 (−0.11, 0.47)^†^
O-level/NVQ2/GCE	0.14 (−0.07, 0.35)	0.14 (−0.07, 0.34)^†^
A-level/NVQ3	0.03 (−0.23, 0.28)	0.05 (−0.21, 0.30)^†^
Higher education/<degree	−0.14 (−0.36, 0.09)	−0.15 (−0.37, 0.08)^†^
Degree/NVQ4/5	Ref	Ref
**Occupation**		
Semi-routine	0.44 (0.30, 0.58)	0.45 (0.31, 0.60)
Lower supervisory/technical	0.32 (0.12, 0.51)	0.30 (0.10, 0.49)
Small employers	0.38 (0.19, 0.58)	0.36 (0.17, 0.56)
Intermediate	0.16 (−0.03, 0.35)	0.19 (−0.00, 0.38)
Managerial/professional	Ref	Ref
**Income fifths**		
1: Lowest	0.42 (0.24, 0.60)	0.39 (0.21, 0.56)
2	0.50 (0.32, 0.69)	0.43 (0.25, 0.62)
3	0.34 (0.15, 0.53)	0.28 (0.09, 0.47)
4	0.08 (−0.11, 0.27)	0.05 (−0.14, 0.25)
5: Highest	Ref	Ref
**Wealth fifths**		
1: Lowest	0.75 (0.58, 0.92)	0.75 (0.58, 0.92)
2	0.52 (0.34, 0.70)	0.51 (0.33, 0.69)
3	0.21 (0.03, 0.40)	0.19 (0.01, 0.38)
4	0.17 (−0.02, 0.36)	0.16 (−0.02, 0.35)
5: Highest	Ref	Ref
**Area-level deprivation fifths**		
5: Most deprived	0.60 (0.42, 0.77)	0.65 (0.48, 0.83)^†^
4	0.37 (0.20, 0.54)	0.41 (0.25, 0.58)^†^
3	0.14 (−0.03, 0.31)	0.16 (−0.01, 0.33)^†^
2	0.21 (0.04, 0.38)	0.22 (0.06, 0.39)^†^
1: Least deprived	Ref	Ref
**Obesity**		
Obesity	0.21 (0.10, 0.32)	0.16 (0.06, 0.27)
Non-obesity	Ref	Ref
BMI per 1 kg/m^2^ increment	0.02 (0.01, 0.03)	0.02 (0.01, 0.03)

BMI, body mass index; CI, confidence interval; CSE, certificate of secondary education; NVQ, National Vocational Qualification; OR, odds ratio; RDCI, rheumatic disease comorbidity index; ref, reference category. SEP indicators adjusted for age and gender. Obesity/BMI adjusted for age, gender, SEP and RDCI. No evidence of interactions (0.08 > *p* < 0.83), except for education and gender (*p* = 0.001) and IMD and gender (*p* = 0.008).

* Regression coefficient is interpreted as: for every one unit increase in the predictors, the outcome will increase/decrease by the regression coefficient.

† As interaction terms between education/area-level deprivation and gender were statistically significant, these estimates are not adjusted for gender; instead, stratified analyses for these are shown in Supplementary Table 4.

For the mobility indicators, stratified analyses showed that the associations were generally stronger for men compared with women (Supplementary Table 2) and for non-obese compared to obese people with OA (Supplementary Table 3). For ADL scores, the associations between lower education, higher deprivation index and more limitations in ADL were stronger for men than women (Supplementary Table 4).

Similar results were found for the sensitivity analyses with imputed data for missing BMI (Supplementary Tables 5, 6).

#### Knee joint replacement surgery

Over a mean follow-up of 4.7 years (SD 2.8), 144 (9.6%) people with symptomatic knee OA reported having at least one knee JRS (8,427 person-years). Education and occupation were not associated with undergoing knee JRS (Table 4). However, those with the lowest income, lowest wealth and highest deprivation index were less likely to undergo knee JRS compared with the highest income, highest wealth and lowest deprivation index [adjusted hazard ratios (adjHRs) 0.64 (95% CI 0.38, 1.06), 0.55 (95% CI 0.33, 0.93), and 0.37 (95% CI 0.19, 0.73), respectively].

**Table 4:** Cox proportional hazard regression for the relationships of socioeconomic indicators and obesity with knee joint replacement surgery.

**Predictors**	**Unadjusted** **HR (95% CI)**	**Adjusted** **HR (95% CI)**
**Education**		
No qualification	0.77 (0.43, 1.37)	0.71 (0.39, 1.28)
Other	1.42 (0.76, 2.68)	1.34 (0.71, 2.55)
NVQ1/CSE	1.23 (0.53, 2.84)	1.17 (0.51, 2.73)
O-level/NVQ2/GCE	0.91 (0.49, 1.70)	0.90 (0.48, 1.68)
A-level/NVQ3	1.05 (0.50, 2.20)	1.06 (0.51, 2.22)
Higher education/<degree	1.28 (0.69, 2.39)	1.25 (0.67, 2.34)
Degree/NVQ4/5	Ref	Ref
**Occupation**		
Semi-routine	0.69 (0.45, 1.05)	0.69 (0.45, 1.06)
Lower supervisory/technical	1.07 (0.63, 1.84)	1.07 (0.62, 1.83)
Small employers	1.03 (0.60, 1.79)	1.03 (0.60, 1.79)
Intermediate	0.80 (0.46, 1.39)	0.79 (0.45, 1.39)
Managerial/professional	Ref	Ref
**Income fifths**		
1: Lowest	0.66 (0.40, 1.09)	0.64 (0.38, 1.06)
2	0.65 (0.39, 1.07)	0.60 (0.36, 1.00)
3	0.74 (0.44, 1.25)	0.70 (0.41, 1.19)
4	0.73 (0.44, 1.23)	0.72 (0.43, 1.21)
5: Highest	Ref	Ref
**Wealth fifths**		
1: Lowest	0.54 (0.32, 0.91)	0.55 (0.33, 0.93)
2	0.52 (0.30, 0.89)	0.52 (0.30, 0.89)
3	0.95 (0.58, 1.56)	0.95 (0.58, 1.55)
4	0.74 (0.44, 1.24)	0.74 (0.44, 1.24)
5: Highest	Ref	Ref
**Index of multiple deprivation fifths**		
5: Most deprived	0.36 (0.18, 0.70)	0.37 (0.19, 0.73)
4	0.80 (0.50, 1.30)	0.83 (0.51, 1.34)
3	0.80 (0.49, 1.30)	0.81 (0.49, 1.31)
2	0.88 (0.56, 1.40)	0.89 (0.56, 1.41)
1: Least deprived	Ref	Ref
**Obesity**		
Obesity	1.56 (1.12, 2.17)	1.87 (1.32, 2.66)
Non-obesity	Ref	Ref
BMI per 1 kg/m^2^ increment	1.05 (1.02, 1.07)	1.07 (1.04, 1.10)

BMI, body mass index; CI, confidence interval; HR, hazard ratio; NVQ, National Vocational Qualification; RDCI, rheumatic disease comorbidity index; ref, reference category. SEP indicators adjusted for age and gender. Obesity/BMI adjusted for age, gender, SEP, RDCI and time-varying HbA1c.

Some indication of interaction for education and gender (*p* = 0.06) and occupation and gender (*p* = 0.07), but not for other SEP indicators (*p* > 0.32). Stratified analyses by gender for education and occupation are shown in Supplementary Table 7. No evidence of interactions between obesity and gender (*p* = 0.961) and SEP indicators (*p* > 0.081).

The interaction terms indicated that the relationships of education and occupation with knee JRS differed by gender. Stratified analyses indicated opposite effect sizes for men and women; for example, adjHRs no qualification vs. degree were 2.00 (95% CI 0.65, 6.14) for men and 0.39 (95% CI 0.19, 0.79) for women (Supplementary Table 7). There was no interaction between obesity and SEP indicators for knee JRS. The results were in line with those of the sensitivity analyses (Supplementary Table 8).

### The associations between obesity and functional limitations and knee joint replacement surgery in people with symptomatic knee OA

#### Functional limitations

Overall, those with obesity had increased risks for limitations in mobility [e.g., for walking 100 yards: adjOR 3.06 (95% CI 2.14, 4.37)] and daily function based on higher ADL scores [adj regression-coefficient 0.16 (95% CI 0.06, 0.27)] compared with those without obesity (Tables 2, 3). There were no gender differences for this association. Similar results were found for the sensitivity analyses (Supplementary Tables 5, 6).

#### Knee joint replacement surgery

Obese people with symptomatic knee OA were more likely to report knee JRS than the non-obese people with OA [adjHR 1.87 (95% CI 1.32, 2.66)] (Table 4). The MFP analysis indicated a linear relationship between BMI and knee JRS fit the data best: the higher the BMI, the higher the hazards for knee JRS [adjHR 1.07 (95% CI 1.04, 1.10)]. There were no gender differences for this association. The results did not differ in the sensitivity analyses (Supplementary Table 8).

### Mediation of obesity for the relationship between lower socioeconomic position and functional limitations

**Figure 1:**
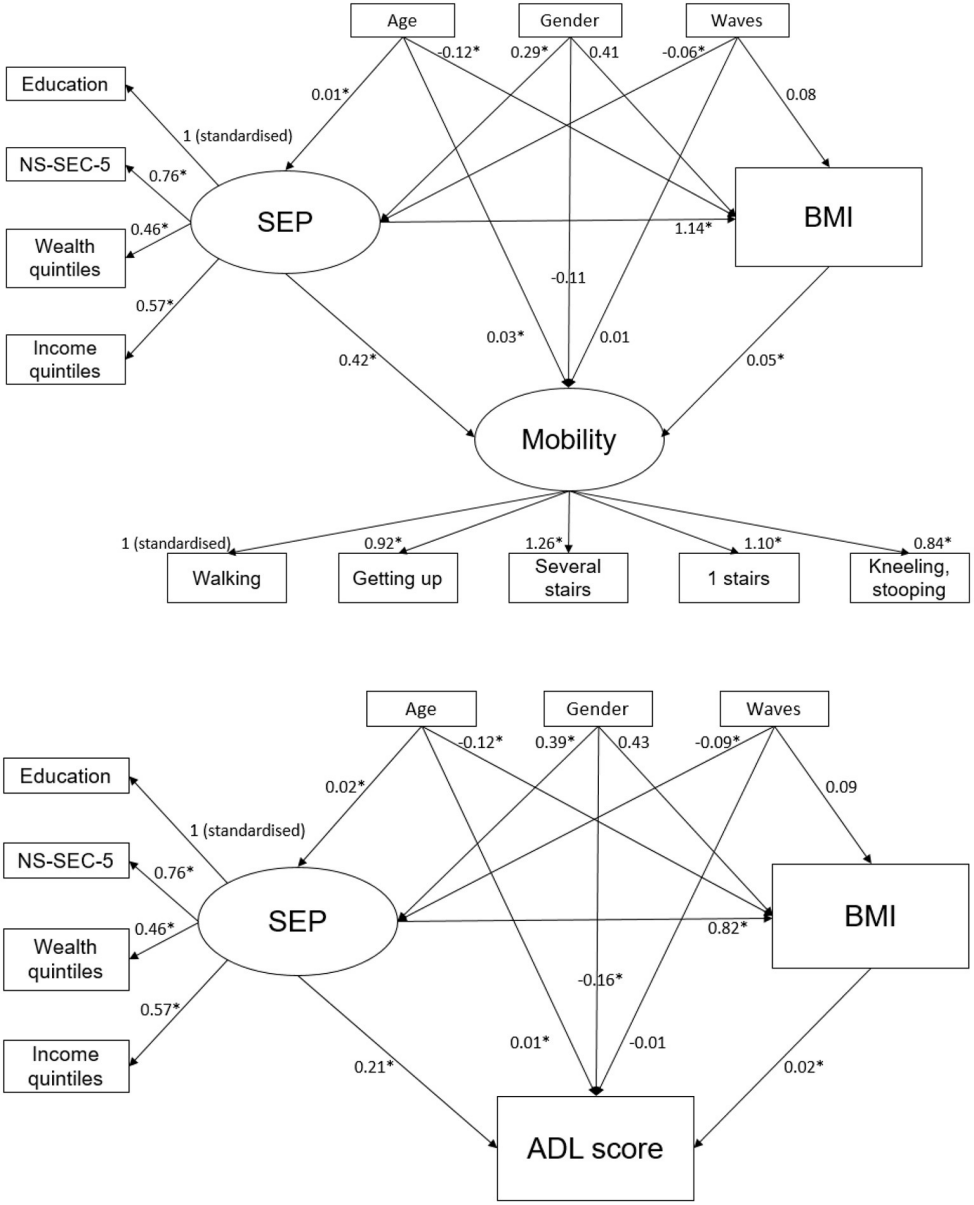
The structural equation models for the relationships between socioeconomic position, BMI and mobility/ADL score, adjusted for age, gender and number of waves attended. *Statistically significant (*p* < 0.05). ADL, activities of daily living; BMI, body mass index; NS-SEC, national statistics socioeconomic classification; SEP, socioeconomic position.

The fit indices of the confirmatory factor analyses and SEMs are shown in Supplementary Table 9. A small proportion of the association between lower SEP and functional limitations was mediated by obesity: 12.5% (95% CI 8.3%, 17.3%) for mobility and 6.2% (95% CI 2.2%, 11.7%) for ADL (Table 5 and Figure 1). Stratified analyses by gender indicated that the proportion mediated by obesity was higher among women [19.4% (95% CI 11.0%, 29.4%) for mobility and 11.7% (95% CI 4.8%, 22.9%) for ADL] compared with men [5.5% (95% CI 1.6%, 10.9%) for mobility and no indirect effect for ADL] (Table 5). As there was no clear association between lower SEP and increased hazards of knee JRS, no mediation analyses were performed for knee JRS as an outcome.

**Table 5:** The total, direct and indirect effect *via* BMI of socioeconomic position as a latent variable on functional limitations (as indicated by difficulties in mobility and activities of daily living) in people with knee OA, adjusted for age and gender.

	**Total**		**Direct**		**Indirect**		**Proportion mediated** (95% CI)^*^
	**β-coefficient (95% CI)**	***p*-value**	**β-coefficient (95% CI)**	***p*-value**	**β-coefficient (95% CI)**	***p*-value**	
**Mobility**							
Total	0.483 (0.394, 0.572)	*p* < 0.001	0.423 (0.336, 0.509)	*p* < 0.001	0.061 (0.038, 0.083)	*p* < 0.001	12.5% (8.3, 17.3%)
Men	0.609 (0.460, 0.758)	*p* < 0.001	0.576 (0.428, 0.723)	*p* < 0.001	0.034 (0.009, 0.058)	*p* = 0.008	5.5% (1.6, 10.9%)
Women	0.400 (0.289, 0.511)	*p* < 0.001	0.322 (0.216, 0.428)	*p* < 0.001	0.078 (0.043, 0.122)	*p* < 0.001	19.4% (11.0, 29.4%)
**Activities of daily living**							
Total	0.224 (0.171, 0.277)	<0.001	0.210 (0.157, 0.264)	<0.001	0.014 (0.004, 0.024)	0.006	6.2% (2.2, 11.7%)
Men	0.292 (0.207, 0.377)	<0.001	0.287 (0.200, 0.374)	<0.001	0.005 (-0.009, 0.019)	0.476	–
Women	0.177 (0.112, 0.243)	<0.001	0.157 (0.091, 0.222)	<0.001	0.021 (0.006, 0.035)	0.007	11.7% (4.8, 22.9%)

CI, confidence interval.

* Calculated by indirect effect/total effect*100%.

95% CI estimated with bootstrapping.

For ADL in men, there was no indirect effect so the proportion mediated was not calculated.

## Discussion

This study indicates that both lower SEP and obesity at baseline were associated with greater odds of functional limitations, measured by mobility and ADL, in people with symptomatic knee OA participating in a large national longitudinal panel study of adults aged ≥50 years in England. A small proportion of the association between lower SEP and functional limitations could be explained by obesity (6.2% for ADL and 12.5% for mobility). Despite this, those with a lower income, lower wealth and higher deprivation were less likely to undergo knee JRS.

In our study among those with symptomatic knee OA a range of SEP indicators were associated with more functional limitations over time. Our findings are consistent with research suggesting that lower SEP is associated with functional limitations in knee and hip OA (9, 10, 32, 33); however, most of these studies were cross-sectional (9, 10, 32) making it difficult to determine the temporal nature of the association. Although the mechanisms are unclear, in our study obesity contributed in part to the association between a lower SEP and functional limitations. However, other factors may also contribute, such as a higher prevalence of comorbidities, lifestyle factors (e.g., physical activity) (34) and local factors (e.g., access to primary care services and less safe places to exercise in deprived areas) (35). There may also be inequalities regarding delivery of care. For example, research has indicated that people with OA with a lower education were less likely to receive advice on exercise compared to those with a higher education (36). Whether these factors mediate the rest of the association between lower SEP and adverse outcomes in symptomatic knee OA should be investigated in future studies.

Similar to our findings, obesity has also been associated with increased functional limitations in people with OA in both cross-sectional (37) and longitudinal studies (38, 39). In general, the relationship between a lower SEP and mobility was stronger for men vs. women; however, a larger proportion of this association was mediated by BMI for women vs. men. This indicates that obesity may be a more important factor leading to mobility limitations for women with lower SEP than men. This might be driven by the relationship between a lower SEP and obesity, which generally appears to be stronger for women than men (22). For men, other factors may play a role, such as occupational exposures: previous studies have found that occupational exposures (i.e., pollution and physically demanding jobs) explained the association between SEP and functional limitations in men but not for women (40). To our knowledge, gender differences for this relationship in OA populations have not been assessed previously.

Although the rates of knee JRS among different educational and occupational groups were similar, the relationships appeared to be gender dependent. In lower educational and occupational groups, women were less likely to have knee JRS and men were more likely to have knee JRS compared to higher educational and occupational groups. For income, wealth and deprivation, the lower fifths were less likely to undergo knee JRS compared to the higher fifths and there were no gender differences observed. Other studies in England (11), Sweden (12) and Denmark (13) also found that there was either an inverse (i.e., those with a lower SEP are less likely to undergo knee JRS) or no relationship between SEP and knee JRS. In general, gender differences have been found previously, where women undergo less knee JRS compared with men despite their potentially greater need (41). Our study adds that the gender differences may be more marked in lower SEP groups.

Given the association between a lower SEP and functional limitations, this may indicate underutilisation of knee JRS in lower SEP groups and specifically in women. Despite free medical care at the point of use in England, there are still socioeconomic inequalities in healthcare (42). Reasons may include that those with lower SEP are less likely to be referred to specialists care (43), fewer clinics and public transport to access clinical appointments and surgery are present in deprived communities (35), and less social support among the lower SEP potentially impacting the willingness to undergo surgery (13). Those with lower SEP may also not be able to take time off work to accommodate the surgery and recovery. Reasons for gender differences have been attributed to women being less willing to undergo surgery (more willing to accept functional decline, less willing to accept the risk of surgery) and specialists are more likely to recommend surgery to men than women (41). Moreover, in line with previous studies (20, 44, 45), our study confirmed the association between obesity and a higher risk of knee JRS. What our study added was that there was no interaction between obesity and SEP indicators for knee JRS; however, this may be because the two factors cancel each other out, i.e., lower SEP associated with lower rates of surgery and obesity with increased rates of surgery.

Strengths of the study include the fact that it was based on a national population sample and included data on serial assessments for up to 16 years. It also included detailed information concerning a range of SEP indicators including education, occupation, income, wealth and area-level deprivation. However, there are a number of limitations that need to be considered in interpreting the findings. The occurrence of OA was based on self-report and therefore subject to errors of recall and potential misclassification. Data from a systematic review including 11 studies comparing OA self-report (at any site) with medical records or American College of Rheumatology criteria, suggest a sensitivity of 0.75 and specificity of 0.89 for self-report (46). We attempted to minimize misclassification by including a requirement for both self-reported diagnosis and self-reported knee pain; however, this does not exclude it. Therefore, caution is required in interpreting the frequency of OA; however, any misclassification is more likely to reduce the chance of finding significant biological associations (bias toward the null). Moreover, the prevalence of self-reported knee OA in our sample was 12.7% (1,804 out of an eligible sample of ELSA of 14,228 in waves 2–8); this is in line with previously reported symptomatic knee OA prevalence estimates in the US of similar age groups [16.7% of people aged ≥45 years in the Johnston County OA project (47); 12.1% of people aged ≥60 years in NHANES III (48)]. Selection bias may have occurred by only including those with a BMI measurement in the main analyses; however, sensitivity analyses where BMI measurements were imputed did not change our findings. Data concerning JRS was also obtained based on self-report, though given the nature of the procedure it seems less likely that this would be subject to errors of recall. Furthermore, JRS data were obtained relatively contemporaneously to the procedure. ADLs and level of mobility are subject to variation over time and possibly prone to recall bias, although our use of data over multiple time points provides a more robust indicator of functional ability over time. In our study, we did not have any information concerning the severity of the underlying OA or its treatment which may have influenced outcome. It is possible, for example, that those with lower SEP may have had more severe disease or were less likely to have therapy and this may in part explain their more severe disability. Finally, our findings were based on a predominantly white English population and caution is needed in generalizing the findings beyond this setting.

Functional limitations are associated with impaired quality of life (49), work productivity (50) and mortality (4) in people with OA. Weight reduction and physical therapy interventions are effective in reducing functional limitations in OA, though there are few data concerning the impact of such interventions in disadvantaged groups for which further research is indicated (51). JRS is effective in relieving pain and improving function in those with knee OA and the lower frequency of surgery in those with lower wealth and living in deprived areas is of concern particularly given the higher levels of disability in these areas. Mediation studies are needed to understand the reasons why those with a lower SEP, and particularly women, are less likely to have JRS even though they appear to have higher disability levels.

To conclude, knee OA in England is expected to rise due to an increase in the number of people with obesity coupled with population ageing. It is important for public health policy to identify predictors of disability and knee JRS. Our results showed that among those with symptomatic knee OA, lower SEP is associated with increased functional limitations and a reduced likelihood of receiving JRS. The increased functional limitations may in part be due to levels of obesity. Further research is required to understand the mechanisms linking lower SEP and adverse outcomes in knee OA and also the reduced likelihood of JRS.

## Data availability statement

Publicly available datasets were analyzed in this study. This anonymized data can be found at: The UK Data Service.

## Ethics statement

Written informed consent was obtained from all participants and ethical approval was acquired from the NHS Research Ethics Committees under the National Research and Ethics Service. The UK Data Service provided anonymized data for this study.

## Author contributions

RW: conception and design, analysis and interpretation of the data, drafting of the article, and final approval of the article. SV, JG, and JH: conception and design, interpretation of the data, critical revision of the article for important intellectual content, final approval of the article, statistical expertise, and obtaining of funding. MC, TO'N, and RC: conception and design, interpretation of the data, critical revision of the article for important intellectual content, and final approval of the article. SV and JH take responsibility for the integrity of the work as a whole, from inception to finished article. All authors contributed to the article and approved the submitted version.
