# Supplementary material for: The association between lower socioeconomic position and functional limitations is partially mediated by obesity in older adults with symptomatic knee osteoarthritis: Findings from the English Longitudinal Study of Ageing
Source: Front Public Health. 2022 Dec 19;10:1053304. doi: 10.3389/fpubh.2022.1053304 (PMC9806847; doi:10.3389/fpubh.2022.1053304)
Supplement: Supplementary file 1 [file Table_1.DOCX]

Supplementary File

### **Table S1: Results of the Schoenfeld test to test proportional hazard assumption**

| **Exposure variables** | **Schoenfeld test (p-value)** |
| --- | --- |
| Education | 0.40 |
| Occupation | 0.55 |
| Income | 0.08 |
| Wealth | 0.71 |
| IMD | 0.81 |
| Obesity | 0.51 |
| BMI | 0.14 |

*A p-value >0.05 indicates that the proportional hazard assumption is met. BMI, body mass index; IMD, index of multiple deprivation; OA, osteoarthritis; RA, rheumatoid arthritis; WC, waist circumference.

###
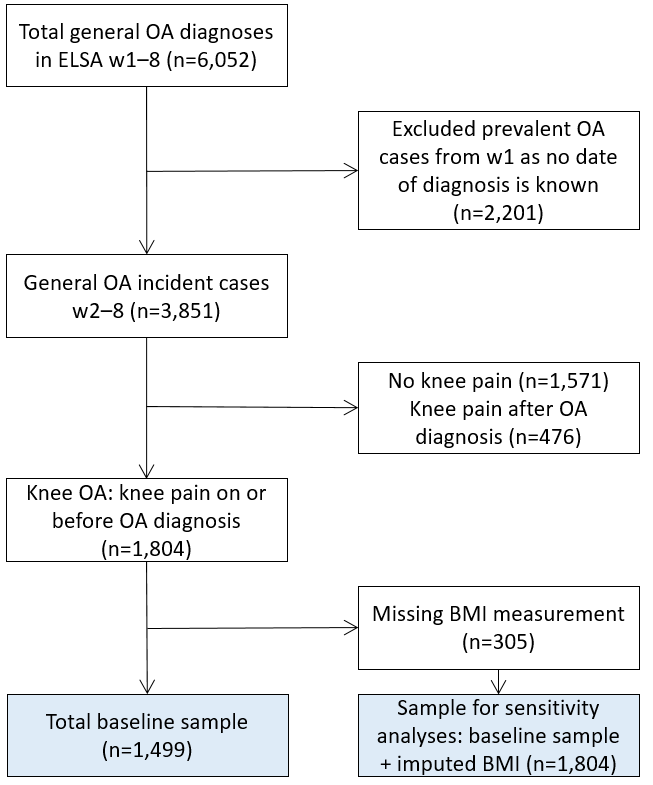
 Figure S1: Flowchart of sample selection for this study

### Table S2: Stratified analyses for the relationships between socioeconomic indicators and difficulties in mobility by gender

| Predictors | Mobility: difficulty (yes) | | | | | | | | | |
| --- | --- | --- | --- | --- | --- | --- | --- | --- | --- | --- |
|  | Walking 100 yards | | Getting up from chair | | Several stairs | | One stair | | Stooping, kneeling, crouching | |
|  | Men (OR (95% CI)) | Women (OR (95% CI)) | Men (OR (95% CI)) | Women (OR (95% CI)) | Men (OR (95% CI)) | Women (OR (95% CI)) | Men (OR (95% CI)) | Women (OR (95% CI)) | Men (OR (95% CI)) | Women (OR (95% CI)) |
| *Education* | | | | | | | | | | |
| No qualification | 20.16 (7.13, 56.99) | 1.42 (0.58, 3.49) | 5.21 (2.75, 9.89) | 2.15 (1.26, 3.37) | 17.53 (7.16, 42.90) | 3.12 (1.60, 6.08) | 17.88 (7.20, 44.38) | 3.49 (1.78, 6.86) | 7.20 (3.68, 14.10) | 1.16 (0.60, 2.22) |
| Other | 3.79 (1.01, 14.25) | 0.55 (0.19, 1.62) | 2.53 (1.12, 5.72) | 1.54 (0.82, 2.88) | 4.86 (1.58, 14.93) | 1.64 (0.75, 3.59) | 8.02 (2.61, 24.61) | 1.28 (0.58, 2.82) | 4.42 (1.88, 10.43) | 0.78 (0.37, 1.67) |
| CSE / NVQ1 | 10.25 (2.77, 37.91) | 0.53 (0.09, 3.04) | 3.84 (1.65, 8.93) | 2.44 (0.90, 6.63) | 11.17 (3.50, 35.68) | 1.40 (0.41, 4.79) | 6.85 (2.21, 21.25) | 1.27 (0.36, 4.46) | 3.86 (1.61, 9.25) | 1.79 (0.52, 6.17) |
| O-level / NVQ2 / GCE | 5.49 (1.75, 17.18) | 1.72 (0.27, 1.91) | 4.22 (2.05, 8.68) | 1.57 (0.89, 2.79) | 5.70 (2.14, 15.16) | 1.59 (0.78, 3.23) | 5.07 (1.88, 13.71) | 1.50 (0.73, 3.09) | 3.90 (1.85, 8.23) | 0.90 (0.45, 1.80) |
| A-level / NVQ3 | 2.47 (0.67, 9.11) | 0.98 (0.27, 3.52) | 1.36 (0.61, 3.05) | 2.62 (1.23, 5.57) | 3.18 (1.07, 9.49) | 2.16 (0.84, 5.52) | 2.96 (0.96, 9.12) | 1.75 (0.69, 4.45) | 2.26 (1.00, 5.11) | 0.79 (0.32, 1.92) |
| Higher education / <degree | 2.97 (0.93, 9.47) | 0.36 (0.12, 1.09) | 1.86 (0.91, 3.81) | 1.65 (0.87, 3.15) | 4.51 (1.70, 11.96) | 1.15 (0.52, 2.55) | 5.14 (1.90, 13.91) | 0.47 (0.20, 1.10) | 3.86 (1.84, 8.12) | 0.58 (0.27, 2.26) |
| Degree / NVQ4/5 | ref | ref | ref | ref | ref | ref | ref | ref | ref | ref |
| *Occupation (NSSEC-5)* | | | | | | | | | | |
| Semi-routine | 8.63 (4.01, 18.57) | 2.84 (1.47, 5.49) | 2.76 (1.68, 4.53) | 1.57 (1.07, 2.30) | 5.71 (2.79, 11.70) | 1.96 (1.20, 3.19) | 7.50 (3.89, 14.46) | 4.26 (2.61, 6.93) | 2.45 (1.44, 4.16) | 1.87 (1.18, 2.96) |
| Lower supervisory / technical | 3.39 (1.41, 8.13) | 3.21 (1.16, 8.92) | 1.58 (0.89, 2.80) | 1.86 (1.00, 3.46) | 2.85 (1.27, 6.36) | 1.78 (0.80, 3.93) | 3.30 (1.57, 6.97) | 3.61 (1.70, 7.68) | 2.45 (1.32, 4.55) | 2.27 (1.04, 4.95) |
| Small employers | 3.88 (1.53, 9.83) | 1.15 (0.42, 3.17) | 2.94 (1.58, 5.47) | 0.98 (0.55, 1.74) | 4.59 (1.95, 10.83) | 0.98 (0.47, 2.04) | 4.13 (1.88, 9.06) | 1.26 (0.60, 2.64) | 2.66 (1.37, 5.17) | 1.26 (0.64, 2.51) |
| Intermediate | 2.24 (0.50, 9.97) | 0.75 (0.32, 1.74) | 1.80 (0.67, 4.84) | 0.82 (0.52, 1.31) | 1.29 (0.34, 4.96) | 1.26 (0.70, 2.27) | 2.19 (0.61, 7.85) | 1.50 (0.82, 2.72) | 0.86 (0.31, 2.40) | 1.29 (0.74, 2.25) |
| Managerial / professional | ref | ref | ref | ref | ref | ref | ref | ref | ref | ref |
| *Income quintiles* | | | | | | | | | | |
| 1: lowest | 10.22 (3.39, 30.77) | 6.24 (2.59, 15.03) | 4.01 (2.05, 7.87) | 1.71 (1.05, 2.77) | 7.51 (2.86, 19.75) | 2.17 (1.19, 3.97) | 5.70 (2.30, 14.10) | 6.87 (3.62, 13.03) | 2.28 (1.10, 4.74) | 1.43 (0.80, 2.53) |
| 2 | 5.28 (1.93, 14.43) | 7.10 (2.87, 17.52) | 2.69 (1.43, 5.06) | 1.69 (1.03, 2.79) | 5.55 (2.33, 13.23) | 3.53 (1.82, 6.84) | 5.72 (2.50, 13.11) | 6.83 (3.54, 13.17) | 2.73 (1.37, 5.42) | 1.70 (0.92, 3.15) |
| 3 | 5.66 (1.92, 16.73) | 5.00 (1.97, 12.74) | 2.20 (1.13, 4.26) | 1.85 (1.09, 3.13) | 3.25 (1.28, 8.24) | 2.57 (1.34, 4.92) | 4.54 (1.84, 11.19) | 4.74 (2.41, 9.31) | 2.13 (1.03, 4.40) | 1.96 (1.04, 3.69) |
| 4 | 1.38 (0.46, 4.13) | 2.41 (0.92, 6.29) | 1.43 (0.73, 2.80) | 1.43 (0.85, 2.43) | 1.71 (0.68, 4.30) | 1.68 (0.87, 3.24) | 1.38 (0.56, 3.40) | 2.95 (1.49, 5.82) | 1.40 (0.69, 2.85) | 1.23 (0.65, 2.31) |
| 5: highest | ref | ref | ref | ref | ref | ref | ref | ref | ref | ref |
| *Wealth quintiles* | | | | | | | | | | |
| 1: lowest | 76.53 (26.17, 223.85) | 22.17 (9.35, 52.58) | 5.04 (2.72, 9.34) | 3.22 (1.98, 5.23) | 23.90 (9.85, 58.00) | 7.51 (4.02, 14.04) | 29.54 (12.29, 70.97) | 18.13 (9.73, 33.77) | 4.84 (2.50, 9.37) | 3.96 (2.17, 7.22) |
| 2 | 23.50 (8.12, 67.99) | 10.97 (4.53, 26.53) | 2.71 (1.44, 5.10) | 2.14 (1.32, 3.46) | 6.05 (2.57, 14.25) | 4.18 (2.23, 7.80) | 10.76 (4.43, 26.08) | 6.14 (3.29, 11.47) | 3.30 (1.69, 6.47) | 2.48 (1.38, 4.46) |
| 3 | 10.30 (3.45, 30.72) | 2.50 (0.99, 6.28) | 2.04 (1.06, 3.93) | 1.50 (0.91, 2.48) | 3.41 (1.39, 8.37) | 2.14 (1.14, 4.04) | 5.50 (2.25, 13.45) | 2.81 (1.46, 5.41) | 3.06 (1.52, 6.15) | 1.30 (0.70, 2.42) |
| 4 | 4.61 (1.50, 14.14) | 2.48 (0.98, 6.26) | 1.36 (0.70, 2.65) | 1.31 (0.80, 2.17) | 2.03 (0.82, 5.05) | 1.96 (1.04, 3.69) | 3.42 (1.36, 8.62) | 1.95 (1.01, 3.76) | 2.74 (1.34, 5.59) | 1.28 (0.70, 2.34) |
| 5: highest | ref | ref | ref | ref | ref | ref | ref | ref | ref | ref |
| *Index of multiple deprivation quintiles* | | | | | | | | | | |
| 5: most deprived | 16.98 (6.20, 46.52) | 8.88 (3.91, 20.14) | 3.91 (2.03, 7.53) | 1.96 (1.22, 3.15) | 11.29 (4.37, 29.17) | 2.40 (1.30, 4.43) | 14.57 (6.17, 34.42) | 6.11 (3.36, 11.12) | 3.19 (1.53, 6.67) | 2.50 (1.39, 4.50) |
| 4 | 5.78 (2.24, 14.90) | 4.24 (1.93, 9.32) | 1.95 (1.07, 3.57) | 1.57 (1.00, 2.46) | 2.98 (1.26, 7.04) | 1.88 (1.04, 3.38) | 4.95 (2.22, 11.04) | 4.16 (2.33, 7.44) | 1.41 (0.72, 2.74) | 1.44 (0.84, 2.49) |
| 3 | 1.86 (0.72, 4.81) | 1.48 (0.66, 3.29) | 0.83 (0.45, 1.52) | 1.23 (0.78, 1.94) | 1.61 (0.69, 3.76) | 1.23 (0.69, 2.20) | 2.37 (1.06, 5.29) | 1.80 (1.00, 3.21) | 0.93 (0.48, 1.78) | 1.56 (0.90, 2.70) |
| 2 | 1.15 (0.44, 3.04) | 2.62 (1.21, 5.65) | 1.25 (0.68, 2.30) | 1.38 (0.89, 2.15) | 1.63 (0.69, 3.83) | 1.63 (0.92, 2.88) | 1.65 (0.73, 3.71) | 2.81 (1.60, 4.93) | 1.46 (0.75, 2.85) | 1.50 (0.88, 2.55) |
| 1: least deprived | ref | ref | ref | ref | ref | ref | ref | ref | ref | ref |

BMI, body mass index; CI, confidence interval; CSE, certificate of secondary education; NS-SEC, National Statistics Socio-economic classification; NVQ, National Vocational Qualification; OR, odds ratio; RDCI, rheumatic disease comorbidity index; ref, reference category. Adjusted for age.

### Table S3: Stratified analyses for the relationships of socioeconomic indicators and difficulties in mobility by obesity status

| Predictors | Mobility: difficulty (yes) | | | | | | | | | |
| --- | --- | --- | --- | --- | --- | --- | --- | --- | --- | --- |
|  | Walking 100 yards | | Getting up from chair | | Several stairs | | One stair | | Stooping, kneeling, crouching | |
|  | Obesity  (OR (95% CI)) | No obesity (OR (95% CI)) | Obesity  (OR (95% CI)) | No obesity (OR (95% CI)) | Obesity  (OR (95% CI)) | No obesity (OR (95% CI)) | Obesity  (OR (95% CI)) | No obesity (OR (95% CI)) | Obesity  (OR (95% CI)) | No obesity (OR (95% CI)) |
| *Education* | | | | | | | | | | |
| No qualification | 2.10 (0.77, 5.73) | 5.76 (2.36, 14.03) | 2.11 (1.17, 3.80) | 3.48 (1.98, 6.14) | 1.77 (0.84, 3.71) | 10.79 (5.27, 22.11) | 3.27 (1.58, 6.78) | 9.75 (4.48, 21.22) | 0.80 (0.38, 1.68) | 4.48 (2.48, 8.11) |
| Other | 0.85 (0.25, 2.86) | 1.68 (0.56, 5.00) | 1.74 (0.86, 3.53) | 1.94 (0.98, 3.84) | 1.67 (0.68, 4.10) | 3.04 (1.29, 7.15) | 2.11 (0.89, 5.04) | 2.79 (1.11, 7.05) | 0.78 (0.32, 1.89) | 2.41 (1.18, 4.92) |
| CSE / NVQ1 | 1.12 (0.27, 4.69) | 2.92 (0.68, 12.53) | 2.54 (1.09, 5.90) | 2.55 (0.98, 6.59) | 2.21 (0.76, 6.43) | 3.34 (1.04, 10.74) | 1.58 (0.56, 4.45) | 2.77 (0.79, 9.64) | 1.00 (0.35, 2.88) | 2.02 (0.75, 5.40) |
| O-level / NVQ2 / GCE | 0.89 (0.30, 2.62) | 2.17 (0.79, 5.95) | 1.76 (0.94, 3.29) | 2.47 (1.31, 4.66) | 1.16 (0.53, 2.54) | 3.55 (1.61, 7.82) | 1.48 (0.68, 3.21) | 3.04 (1.28, 7.22) | 0.57 (0.26, 1.26) | 2.85 (1.47, 5.52) |
| A-level / NVQ3 | 1.19 (0.32, 4.39) | 0.93 (0.26, 3.39) | 1.96 (0.91, 4.22) | 1.54 (0.71, 3.35) | 1.51 (0.58, 3.95) | 2.36 (0.89, 6.24) | 1.88 (0.74, 4.78) | 1.53 (0.52, 4.49) | 0.79 (0.30, 2.04) | 1.26 (0.57, 2.78) |
| Higher education / <degree | 0.54 (0.16, 1.84) | 1.34 (0.48, 3.79) | 1.59 (0.79, 3.23) | 1.81 (0.95, 3.47) | 0.82 (0.34, 1.98) | 3.55 (1.59, 7.96) | 1.02 (0.42, 2.45) | 1.56 (0.63, 3.83) | 0.45 (0.19, 1.08) | 2.63 (1.35, 5.14) |
| Degree /NVQ4/5 | ref | ref | ref | ref | ref | ref | ref | ref | ref | ref |
| *Occupation (NSSEC-5)* | | | | | | | | | | |
| Semi-routine | 3.58 (1.74, 7.36) | 3.81 (1.96, 7.39) | 1.76 (1.16, 2.66) | 1.79 (1.16, 2.76) | 1.94 (1.15, 3.27) | 2.82 (1.58, 5.03) | 4.22 (2.53, 7.03) | 4.81 (2.68, 8.63) | 1.50 (0.91, 2.46) | 2.18 (1.37, 3.48) |
| Lower supervisory / technical | 1.88 (0.73, 4.83) | 3.29 (1.29, 8.41) | 1.26 (0.73, 2.19) | 1.59 (0.84, 3.00) | 1.16 (0.59, 2.30) | 2.24 (0.96, 5.24) | 2.67 (1.38, 5.19) | 2.47 (1.09, 5.63) | 1.46 (0.74, 2.88) | 2.53 (1.28, 5.01) |
| Small employers | 2.30 (0.79, 6.68) | 1.91 (0.80, 4.56) | 1.82 (0.97, 3.39) | 1.56 (0.89, 2.76) | 1.72 (0.79, 3.76) | 2.31 (1.09, 4.88) | 2.24 (1.06, 4.76) | 2.19 (1.05, 4.58) | 1.46 (0.68, 3.12) | 2.09 (1.14, 3.82) |
| Intermediate | 1.36 (0.50, 3.73) | 0.72 (0.28, 1.83) | 0.96 (0.54, 1.70) | 0.95 (0.53, 1.69) | 1.17 (0.57, 2.38) | 1.54 (0.73, 3.24) | 1.91 (0.96, 3.80) | 1.28 (0.59, 2.79) | 1.35 (0.68, 2.68) | 1.09 (0.60, 2.00) |
| Managerial / professional | ref | ref | ref | ref | ref | ref | ref | ref | ref | ref |
| *Income quintiles* | | | | | | | | | | |

| 1: lowest | 8.44 (3.26, 21.83) | 5.00 (1.97, 12.72) | 2.33 (1.39, 3.91) | 1.94 (1.10, 3.41) | 2.08 (1.08, 3.98) | 3.73 (1.83, 7.63) | 6.65 (3.48, 12.71) | 4.84 (2.19, 10.69) | 1.78 (0.63, 2.22) | 2.05 (1.13, 3.72) |
| --- | --- | --- | --- | --- | --- | --- | --- | --- | --- | --- |
| 2 | 7.13 (2.73, 18.61) | 4.80 (1.96, 11.76) | 1.97 (1.17, 3.32) | 1.98 (1.13, 3.48) | 1.77 (0.91, 3.46) | 7.73 (3.72, 16.05) | 5.44 (2.83, 10.44) | 6.73 (3.14, 14.43) | 1.30 (0.68, 2.48) | 2.82 (1.55, 5.14) |
| 3 | 5.60 (2.10, 14.96) | 3.90 (1.48, 10.31) | 1.60 (0.94, 2.73) | 2.21 (1.22, 4.02) | 1.72 (0.88, 3.34) | 3.41 (1.61, 7.22) | 4.23 (2.16, 8.30) | 4.14 (1.84, 9.32) | 1.24 (0.65, 2.36) | 2.66 (1.42, 5.01) |
| 4 | 2.54 (0.88, 7.36) | 1.34 (0.51, 3.56) | 1.69 (0.96, 2.98) | 1.19 (0.66, 2.16) | 1.59 (0.78, 3.27) | 1.76 (0.85, 3.65) | 2.64 (1.31, 5.30) | 1.73 (0.76, 3.95) | 1.13 (0.57, 2.23) | 1.45 (0.77, 2.71) |
| 5: highest | ref | ref | ref | ref | ref | ref | ref | ref | ref | ref |
| *Wealth quintiles* | | | | | | | | | | |
| 1: lowest | 24.62 (9.03, 67.18) | 25.89 (10.63, 63.07) | 2.86 (1.66, 4.93) | 3.60 (2.10, 6.16) | 3.74 (1.85, 7.59) | 13.75 (6.63, 28.51) | 11.18 (5.61, 22.28) | 24.26 (11.48, 51.27) | 2.63 (1.33, 5.18) | 3.86 (2.12, 7.04) |
| 2 | 7.41 (2.69, 20.42) | 18.15 (7.51, 43.89) | 1.50 (0.86, 2.64) | 2.84 (1.68, 4.81) | 1.37 (0.68, 2.79) | 7.88 (3.92, 15.83) | 4.11 (2.04, 8.29) | 9.34 (4.51, 19.33) | 1.26 (0.63, 2.52) | 3.75 (2.10, 6.71) |
| 3 | 3.42 (1.20, 9.76) | 3.50 (1.42, 8.61) | 1.41 (0.79, 2.50) | 1.65 (0.95, 2.87) | 0.93 (0.45, 1.93) | 3.62 (1.80, 7.30) | 2.61 (1.26, 5.41) | 3.30 (1.58, 6.90) | 1.14 (0.56, 2.32) | 1.81 (1.00, 3.29) |
| 4 | 2.25 (0.75, 6.76) | 2.96 (1.19, 7.38) | 1.41 (0.78, 2.54) | 1.05 (0.60, 1.82) | 0.95 (0.45, 2.04) | 2.25 (1.11, 4.58) | 1.88 (0.88, 4.00) | 2.07 (0.96, 4.45) | 1.11 (0.54, 2.29) | 1.77 (0.98, 3.18) |
| 5: highest | ref | ref | ref | ref | ref | ref | ref | ref | ref | ref |
| *Index of multiple deprivation quintiles* | | | | | | | | | | |
| 5: most deprived | 10.15 (4.31, 23.90) | 11.44 (4.74, 27.61) | 2.14 (1.31, 3.50) | 2.85 (1.60, 5.09) | 2.17 (1.15, 4.08) | 6.76 (3.11, 14.68) | 4.91 (2.69, 8.95) | 13.72 (6.42, 29.32) | 3.37 (1.81, 6.26) | 2.17 (1.15, 4.11) |
| 4 | 5.76 (2.49, 13.31) | 3.79 (1.67, 8.59) | 1.85 (1.14, 2.98) | 1.54 (0.91, 2.61) | 1.44 (0.78, 2.66) | 3.02 (1.50, 6.11) | 3.71 (2.05, 6.70) | 4.98 (2.47, 10.05) | 1.74 (0.98, 3.11) | 1.23 (0.69, 2.19) |
| 3 | 2.14 (0.91, 5.02) | 1.21 (0.53, 2.75) | 1.26 (0.78, 2.06) | 0.91 (0.54, 1.54) | 1.08 (0.58, 2.01) | 1.64 (0.83, 3.24) | 2.07 (1.14, 3.78) | 1.81 (0.90, 3.64) | 1.44 (0.80, 2.57) | 1.14 (0.64, 2.02) |
| 2 | 3.89 (1.66, 9.10) | 1.05 (0.47, 2.33) | 1.82 (1.11, 2.97) | 1.07 (0.65, 1.78) | 1.72 (0.92, 3.23) | 1.71 (0.88, 3.33) | 3.04 (1.67, 5.53) | 1.88 (0.95, 3.69) | 2.07 (1.14, 3.74) | 1.29 (0.74, 2.26) |
| 1: least deprived | ref | ref | ref | ref | ref | ref | ref | ref | ref | ref |

BMI, body mass index; CI, confidence interval; CSE, certificate of secondary education; NS-SEC, National Statistics Socio-economic classification; NVQ, National Vocational Qualification; OR, odds ratio; RDCI, rheumatic disease comorbidity index; ref, reference category. Adjusted for age and gender.

### Table S4: Stratified analysis for the relationships of education and deprivation with difficulties in activities of daily living scores (0–6, 0 = no difficulties) by gender

| Predictors | Age-adjusted regression coefficient (95% CI) | |
| --- | --- | --- |
|  | Men | Women |
| *Education* | | |
| No qualification | 0.69 (0.42, 0.97) | 0.03 (-0.22, 0.28) |
| Other | 0.26 (-0.10, 0.62) | -0.16 (-0.45, 0.14) |
| CSE / NVQ1 | 0.41 (0.05, 0.78) | -0.04 (-0.51, 0.42) |
| O-level / NVQ2 / GCE | 0.37 (0.06, 0.69) | -0.04 (-0.31, 0.23) |
| A-level / NVQ3 | 0.10 (-0.26, 0.45) | 0.02 (-0.34, 0.37) |
| Higher education / <degree | 0.08 (-0.24, 0.39) | -0.34 (-0.65, -0.03) |
| Degree / NVQ4/5 | ref | ref |
| *Index of multiple deprivation quintiles* | | |
| 5: most deprived | 0.85 (0.57, 1.13) | 0.53 (0.31, 0.75) |
| 4 | 0.39 (0.13, 0.66) | 0.41 (0.20, 0.63) |
| 3 | 0.12 (-0.15, 0.39) | 0.17 (-0.04, 0.39) |
| 2 | -0.04 (-0.31, 0.23) | 0.37 (0.16, 0.58) |
| 1: least deprived | ref | Ref |

CI, confidence interval; CSE, certificate of secondary education; NVQ, National Vocational Qualification; ref, reference category.

### Table S5: Sensitivity analysis – random-effect generalised linear mixed models for the relationships of socioeconomic indicators and obesity with difficulties in mobility

| Predictors | Mobility: difficulty (yes) | | | | | | | | | |
| --- | --- | --- | --- | --- | --- | --- | --- | --- | --- | --- |
|  | Walking 100 yards | | Getting up from chair | | Several stairs | | One stair | | Stooping, kneeling, crouching | |
|  | Unadjusted (OR (95% CI)) | Adjusted (OR (95% CI)) | Unadjusted (OR (95% CI)) | Adjusted (OR (95% CI)) | Unadjusted (OR (95% CI)) | Adjusted (OR (95% CI)) | Unadjusted (OR (95% CI)) | Adjusted (OR (95% CI)) | Unadjusted (OR (95% CI)) | Adjusted (OR (95% CI)) |
| *Education* | | | | | | | | | | |
| No qualification | 7.89 (4.04, 15.42) | 4.69 (2.37, 9.27) | 2.85 (1.95, 4.14) | 2.82 (1.92, 4.13) | 7.79 (4.72, 12.85) | 5.56 (3.39, 9.10) | 9.69 (5.72, 16.49) | 6.77 (4.03, 11.36) | 3.21 (2.10, 4.91) | 2.87 (1.87, 4.41) |
| Other | 1.54 (0.67, 3.53) | 1.41 (0.61, 3.25) | 1.74 (1.09, 2.76) | 1.72 (1.08, 2.74) | 2.72 (1.47, 5.02) | 2.18 (1.19, 3.97) | 2.99 (1.58, 5.64) | 2.39 (1.28, 4.47) | 2.04 (1.21, 3.44) | 1.91 (1.13, 3.23) |
| CSE / NVQ1 | 3.67 (1.31, 10.28) | 2.62 (0.92, 7.46) | 2.62 (1.45, 4.74) | 2.66 (1.47, 4.82) | 3.49 (1.59, 7.63) | 3.53 (1.65, 7.58) | 3.51 (1.58, 7.80) | 3.12 (1.43, 6.82) | 2.24 (1.15, 4.38) | 2.18 (1.12, 4.27) |
| O-level / NVQ2 / GCE | 1.82 (0.87, 3.81) | 1.78 (0.84, 3.78) | 2.07 (1.37, 3.14) | 2.06 (1.36, 3.12) | 2.51 (1.46, 4.31) | 2.29 (1.35, 3.89) | 2.37 (1.33, 4.21) | 2.23 (1.27, 3.92) | 1.97 (1.24, 3.14) | 1.92 (1.21, 3.06) |
| A-level / NVQ3 | 1.06 (0.42, 2.67) | 1.43 (0.57, 3.61) | 1.64 (0.98, 2.73) | 1.64 (0.98, 2.74) | 1.73 (0.88, 3.40) | 1.88 (0.97, 3.63) | 1.72 (0.85, 3.47) | 1.85 (0.93, 3.68) | 1.25 (0.71, 2.21) | 1.28 (0.72, 2.25) |
| Higher education / <degree | 1.17 (0.52, 2.62) | 0.96 (0.42, 2.17) | 1.73 (1.10, 2.72) | 1.73 (1.10, 2.72) | 1.90 (1.06, 3.41) | 1.85 (1.04, 3.27) | 1.52 (0.81, 2.84) | 1.43 (0.77, 2.66) | 1.70 (1.03, 2.82) | 1.67 (1.01, 2.76) |
| Degree / NVQ4/5 | ref | ref | ref | ref | ref | ref | ref | ref | ref | ref |
| *Occupation (NSSEC-5)* | | | | | | | | | | |
| Semi-routine | 5.25 (3.20, 8.61) | 5.30 (3.26, 8.62) | 2.02 (1.52, 2.69) | 1.99 (1.49, 2.64) | 3.36 (2.29, 4.93) | 2.92 (2.01, 4.25) | 5.34 (3.62, 7.86) | 5.05 (3.47, 7.35) | 2.18 (1.58, 3.01) | 2.11 (1.53, 2.91) |
| Lower supervisory / technical | 3.63 (1.83, 7.20) | 3.30 (1.69, 6.43) | 1.55 (1.05, 2.30) | 1.56 (1.05, 2.31) | 1.72 (1.00, 2.96) | 1.81 (1.07, 3.06) | 2.90 (1.68, 5.01) | 2.87 (1.69, 4.85) | 2.08 (1.30, 3.32) | 2.08 (1.30, 3.31) |
| Small employers | 2.77 (1.40, 5.49) | 2.40 (1.23, 4.69) | 1.72 (1.17, 2.55) | 1.72 (1.16, 2.54) | 1.85 (1.09, 3.15) | 1.79 (1.07, 2.99) | 2.50 (1.47, 4.26) | 2.31 (1.38, 3.86) | 1.86 (1.18, 2.92) | 1.83 (1.17, 2.88) |
| Intermediate | 0.97 (0.50, 1.91) | 1.00 (0.51, 1.96) | 1.00 (0.69, 1.46) | 0.96 (0.66, 1.41) | 2.05 (1.24, 3.37) | 1.54 (0.94, 2.53) | 1.85 (1.11, 3.09) | 1.64 (0.99, 2.72) | 1.48 (0.97, 2.26) | 1.37 (0.89, 2.11) |
| Managerial / professional | ref | ref | ref | ref | ref | ref | ref | ref | ref | ref |

| *Income quintiles* | | | | | | | | | | |
| --- | --- | --- | --- | --- | --- | --- | --- | --- | --- | --- |
| 1: lowest | 9.50 (4.68, 19.29) | 7.77 (3.84, 15.72) | 2.21 (1.51, 2.25) | 2.18 (1.48, 3.21) | 3.79 (2.26, 6.36) | 2.97 (1.78, 4.96) | 8.07 (4.68, 13.91) | 6.42 (3.74, 10.99) | 1.84 (1.19, 2.85) | 1.70 (1.10, 2.64) |
| 2 | 9.90 (5.02, 19.49) | 6.59 (3.38, 12.85) | 1.93 (1.30, 2.86) | 1.91 (1.28, 2.86) | 4.75 (2.82, 8.03) | 3.77 (2.23, 6.39) | 8.37 (4.89, 14.32) | 6.07 (3.58, 10.29) | 2.27 (1.47, 3.50) | 2.06 (1.32, 3.21) |
| 3 | 8.91 (4.45, 17.84) | 6.22 (3.14, 12.31) | 1.92 (1.31, 2.82) | 1.90 (1.29, 2.81) | 3.32 (1.98, 5.59) | 2.58 (1.54, 4.32) | 6.44 (3.79, 10.95) | 4.68 (2.78, 7.88) | 2.20 (1.40, 3.47) | 2.01 (1.26, 3.18) |
| 4 | 2.10 (1.05, 4.22) | 1.85 (0.93, 3.68) | 1.31 (0.88, 1.94) | 1.31 (0.88, 1.94) | 1.58 (0.93, 2.68) | 1.50 (0.89, 2.53) | 2.34 (1.37, 4.02) | 2.16 (1.27, 3.67) | 1.38 (0.88, 2.17) | 1.35 (0.86, 2.11) |
| 5: highest | ref | ref | ref | ref | ref | ref | ref | ref | ref | ref |
| *Wealth quintiles* | | | | | | | | | | |
| 1: lowest | 38.25 (19.00, 77.01) | 37.50 (19.09, 73.65) | 3.60 (2.51, 5.17) | 3.58 (2.49, 5.14) | 10.94 (6.68, 17.93) | 10.33 (6.41, 16.65) | 19.48 (11.60, 32.69) | 18.71 (11.37, 30.80) | 4.53 (3.01, 6.82) | 4.44 (2.95, 6.67) |
| 2 | 16.62 (8.23, 33.57) | 15.30 (7.67, 30.49) | 2.41 (1.64, 3.52) | 2.38 (1.63, 3.49) | 4.77 (2.88, 7.90) | 4.31 (2.63, 7.05) | 7.76 (4.58, 13.17) | 6.93 (4.13, 11.62) | 2.92 (1.91, 4.46) | 2.82 (1.85, 4.32) |
| 3 | 4.94 (2.38, 10.25) | 4.24 (2.11, 8.52) | 1.69 (1.16, 2.46) | 1.66 (1.14, 2.43) | 2.53 (1.52, 4.20) | 2.21 (1.35, 3.60) | 3.48 (2.03, 5.97) | 3.00 (1.79, 5.03) | 1.91 (1.21, 2.99) | 1.81 (1.56, 2.83) |
| 4 | 3.44 (1.63, 7.25) | 3.19 (1.56, 6.54) | 1.35 (0.92, 1.97) | 1.33 (0.91, 1.95) | 1.98 (1.19, 3.31) | 1.79 (1.10, 2.92) | 2.24 (1.30, 3.86) | 2.05 (1.22, 3.44) | 1.74 (1.14, 2.66) | 1.69 (1.11, 2.58) |
| 5: highest | ref | ref | ref | ref | ref | ref | ref | ref | ref | ref |
| *Index of multiple deprivation quintiles* | | | | | | | | | | |
| 5: most deprived | 10.11 (5.42, 18.88) | 14.20 (7.68, 26.24) | 2.42 (1.69, 3.45) | 2.50 (1.75, 3.57) | 3.66 (2.23, 6.00) | 4.56 (2.82, 7.39) | 6.78 (4.18, 11.02) | 9.07 (5.66, 14.54) | 2.61 (1.71, 3.99) | 2.85 (1.87, 4.35) |
| 4 | 4.15 (2.27, 7.59) | 5.18 (2.87, 9.33) | 1.68 (1.19, 2.36) | 1.72 (1.22, 2.41) | 1.95 (1.22, 3.11) | 2.29 (1.45, 3.61) | 3.64 (2.28, 5.83) | 4.48 (2.85, 7.06) | 1.43 (0.96, 2.12) | 1.52 (1.03, 2.25) |
| 3 | 1.48 (0.80, 2.72) | 1.66 (0.92, 3.02) | 0.95 (0.68, 1.34) | 0.97 (0.69, 1.36) | 1.18 (0.74, 1.88) | 1.31 (0.84, 2.05) | 1.56 (0.97, 2.50) | 1.76 (1.12, 2.78) | 1.23 (0.83, 1.82) | 1.27 (0.86, 1.88) |
| 2 | 1.94 (1.07, 3.55) | 2.12 (1.18, 3.80) | 1.36 (0.97, 1.91) | 1.37 (0.98, 1.92) | 1.42 (0.89, 2.25) | 1.50 (0.96, 2.34) | 2.06 (1.30, 3.29) | 2.23 (1.42, 3.48) | 1.44 (0.98, 2.13) | 1.47 (1.00, 2.17) |
| 1: least deprived | ref | ref | ref | ref | ref | ref | ref | ref | ref | ref |

| *Obesity* | | | | | | | | | | |
| --- | --- | --- | --- | --- | --- | --- | --- | --- | --- | --- |
| Obesity | 3.23 (2.13, 4.88) | 2.87 (1.96, 4.20) | 1.90 (1.52, 2.38) | 1.64 (1.31, 2.04) | 3.33 (2.41, 4.62) | 2.88 (2.13, 3.90) | 2.87 (2.10, 3.94) | 2.52 (1.89, 3.34) | 2.50 (1.92, 3.25) | 2.21 (1.70, 2.87) |
| Non-obesity | ref | ref | ref | ref | ref | ref | ref | ref | ref | ref |
| BMI per 1 kg/m^2^ increment | 1.12 (1.09, 1.16) | 1.12 (1.09, 1.15) | 1.07 (1.05, 1.09) | 1.06 (1.04, 1.08) | 1.13 (1.10, 1.16) | 1.12 (1.09, 1.15) | 1.10 (1.08, 1.13) | 1.10 (1.07, 1.12) | 1.11 (1.08, 1.13) | 1.10 (1.07, 1.12) |

BMI, body mass index; CI, confidence interval; CSE, certificate of secondary education; NS-SEC, National Statistics Socio-economic classification; NVQ, National Vocational Qualification; OR, odds ratio; RDCI, rheumatic disease comorbidity index; ref, reference category. SEP indicators adjusted for age and gender. Obesity/BMI adjusted for age, gender, SEP and RDCI.

### Table S6: Sensitivity analysis – random-effect linear mixed models for the relationships of socioeconomic indicators and obesity with difficulties in activities in daily living score (0–6, 0= no difficulties)

| Predictors | Unadjusted  Regression coefficient (95% CI) | Adjusted  Regression coefficient (95% CI) |
| --- | --- | --- |
| *Education* | | |
| No qualification | 0.43 (0.24, 0.61) | 0.36 (0.17, 0.54) |
| Other | 0.09 (-0.15, 0.33) | 0.06 (-0.18, 0.29) |
| CSE / NVQ1 | 0.25 (-0.04, 0.55) | 0.19 (-0.10, 0.49) |
| O-level / NVQ2 / GCE | 0.14 (-0.07, 0.35) | 0.14 (-0.07, 0.35) |
| A-level / NVQ3 | -0.01 (-0.26, 0.25) | 0.01 (-0.25, 0.26) |
| Higher education / <degree | -0.06 (-0.29, 0.17) | -0.08 (-0.31, 0.15) |
| Degree / NVQ4/5 | ref | ref |
| *Occupation (NSSEC-5)* | | |
| Semi-routine | 0.42 (0.29, 0.56) | 0.43 (0.29, 0.57) |
| Lower supervisory/technical | 0.36 (0.16, 0.56) | 0.34 (0.14, 0.54) |
| Small employers | 0.32 (0.13, 0.52) | 0.30 (0.11, 0.49) |
| Intermediate | 0.12 (-0.07, 0.31) | 0.14 (-0.05, 0.33) |
| Managerial / professional | ref | ref |
| *Income quintiles* | | |
| 1: lowest | 0.44 (0.25, 0.64) | 0.40 (0.21, 0.60) |
| 2 | 0.54 (0.36, 0.73) | 0.46 (0.27, 0.65) |
| 3 | 0.38 (0.18, 0.57) | 0.31 (0.11, 0.50) |
| 4 | 0.05 (-0.14, 0.25) | 0.03 (-0.17, 0.23) |
| 5: highest | ref | ref |
| *Wealth quintiles* | | |
| 1: lowest | 0.78 (0.59, 0.96) | 0.77 (0.59, 0.95) |
| 2 | 0.56 (0.38, 0.75) | 0.55 (0.37, 0.73) |
| 3 | 0.22 (0.03, 0.41) | 0.19 (-0.00, 0.37) |
| 4 | 0.23 (0.04, 0.42) | 0.21 (0.03, 0.40) |
| 5: highest | ref | ref |
| *Index of multiple deprivation quintiles* | | |
| 5: most deprived | 0.68 (0.50, 0.85) | 0.75 (0.57, 0.92) |
| 4 | 0.36 (0.19, 0.53) | 0.40 (0.23, 0.57) |
| 3 | 0.13 (-0.04, 0.30) | 0.15 (-0.02, 0.32) |
| 2 | 0.26 (0.09, 0.43) | 0.27 (0.10, 0.44) |
| 1: least deprived | ref | ref |
| *Obesity* | | |
| Obesity | 0.21 (0.08, 0.34) | 0.17 (0.04, 0.30) |
| Non-obesity | ref | ref |
| BMI per 1 kg/m^2^ increment | 0.02 (0.01, 0.03) | 0.02 (0.01, 0.03) |

BMI, body mass index; CI, confidence interval; CSE, certificate of secondary education; NS-SEC, National Statistics Socio-economic classification; NVQ, National Vocational Qualification; OR, odds ratio; RDCI, rheumatic disease comorbidity index; ref, reference category. SEP indicators adjusted for age and gender. Obesity/BMI adjusted for age, gender, SEP and RDCI.

### Table S7: Stratified analyses for the relationships of education and occupation with knee joint replacement surgery by gender

|  | Men  Age-adjusted HR (95% CI) | Women Age-adjusted HR (95% CI) |
| --- | --- | --- |
| *Education* | | |
| No qualification | 2.00 (0.65, 6.14) | 0.39 (0.19, 0.79) |
| Other | 3.36 (1.01, 11.17) | 0.79 (0.37, 1.71) |
| CSE / NVQ1 | 2.56 (0.68, 9.61) | 0.73 (0.21, 2.58) |
| O-level / NVQ2 / GCE | 1.56 (0.44, 5.53) | 0.64 (0.31, 1.31) |
| A-level / NVQ3 | 2.56 (0.72, 9.10) | 0.62 (0.24, 1.63) |
| Higher education / <degree | 3.11 (0.99, 9.79) | 0.72 (0.33, 1.55) |
| Degree / NVQ4/5 | ref | ref |
| *Occupation* | | |
| Semi-routine | 1.12 (0.55, 2.25) | 0.53 (0.31, 0.91) |
| Lower supervisory/technical | 1.12 (0.51, 2.46) | 1.05 (0.49, 2.23) |
| Small employers | 1.40 (0.64, 3.04) | 0.79 (0.36, 1.74) |
| Intermediate | n/a* | 0.81 (0.45, 1.48) |
| Managerial/ professional | ref | ref |

CI, confidence interval; CSE, certificate of secondary education; HR, hazard ratio; NS-SEC, National Statistics Socio-economic classification; NVQ, National Vocational Qualification. *No knee JRS in this group.

### Table S8: Sensitivity analysis – Cox regression analysis for the relationships of socioeconomic indicators and obesity with knee joint replacement surgery

| Predictors | Unadjusted  HR (95% CI) | Adjusted  HR (95% CI) |
| --- | --- | --- |
| *Education* | | |
| No qualification | 0.71 (0.41, 1.23) | 0.64 (0.37, 1.13) |
| Other | 1.47 (0.81, 2.66) | 1.39 (0.76, 2.53) |
| CSE/NVQ1 | 1.05 (0.46, 2.39) | 1.01 (0.44, 2.31) |
| O-level/NVQ2/ GCE | 0.87 (0.48, 1.58) | 0.86 (0.47, 1.56) |
| A-level NVQ3 | 0.93 (0.45, 1.91) | 0.94 (0.46, 1.94) |
| Higher education/<degree | 1.36 (0.76, 2.43) | 1.32 (0.74, 2.37) |
| Degree/NVQ4/5 | ref | ref |
| *Occupation (NSSEC-5)* | | |
| Semi-routine | 0.72 (0.48, 1.08) | 0.71 (0.47, 1.07) |
| Lower supervisory/technical | 1.13 (0.68, 1.89) | 1.12 (0.67, 1.88) |
| Small employers | 1.09 (0.65, 1.83) | 1.09 (0.65, 1.83) |
| Intermediate | 0.80 (0.47, 1.35) | 0.78 (0.45, 1.33) |
| Managerial/ professional | ref | ref |
| *Income quintiles* | | |
| 1: lowest | 0.65 (0.40, 1.07) | 0.63 (0.38, 1.04) |
| 2 | 0.68 (0.41, 1.14) | 0.63 (0.38, 1.07) |
| 3 | 0.73 (0.43, 1.25) | 0.69 (0.40, 1.18) |
| 4 | 0.76 (0.45, 1.29) | 0.76 (0.45, 1.28) |
| 5: highest | ref | ref |
| *Wealth quintiles* | | |
| 1: lowest | 0.53 (0.31, 0.88) | 0.53 (0.32, 0.89) |
| 2 | 0.55 (0.32, 0.95) | 0.55 (0.32, 0.95) |
| 3 | 1.06 (0.65, 1.72) | 1.04 (0.64, 1.70) |
| 4 | 0.83 (0.50, 1.37) | 0.83 (0.50, 1.37) |
| 5: highest | ref | ref |
| *Index of multiple deprivation quintiles* | | |
| 5: most deprived | 0.32 (0.16, 0.61) | 0.33 (0.17, 0.64) |
| 4 | 0.78 (0.49, 1.24) | 0.80 (0.50, 1.28) |
| 3 | 0.87 (0.55, 1.37) | 0.87 (0.55, 1.38) |
| 2 | 0.89 (0.57, 1.38) | 0.89 (0.57, 1.39) |
| 1: least deprived | ref | ref |
| *Obesity* | | |
| Obesity | 1.50 (1.06, 2.13) | 1.81 (1.30, 2.51) |
| Non-obesity | ref | ref |
| BMI per 1 kg/m^2^ increment | 1.04 (1.02, 1.07) | 1.07 (1.04, 1.10) |

BMI, body mass index; CI, confidence interval; cm, centimetres; CSE, certificate of secondary education; HR, hazard ratio; NS-SEC, National Statistics Socio-economic classification; NVQ, National Vocational Qualification; RDCI, rheumatic disease comorbidity index; ref, reference category. SEP indicators adjusted for age and gender. Obesity/BMI adjusted for age, gender, SEP, RDCI and time-varying HbA1c.

### Table S9: Fit indices of the structural equation models

|  | CFI | RMSEA | SRMSR |
| --- | --- | --- | --- |
| *Model fit of latent variables* | | | |
| SEP | 0.998 | 0.039 | 0.007 |
| Mobility | 0.994 | 0.064 | 0.016 |
| *Model fit for structural equation models* | | | |
| ADL | 0.931 | 0.071 | 0.035 |
| Mobility | 0.941 | 0.060 | 0.040 |

CFA, confirmatory factor analysis; CFI, comparative fit index; OA, osteoarthritis; RA, rheumatoid arthritis; RMSEA, root mean square error of approximation; SEP, socioeconomic position; SRMSR, standardised root mean square residual.
